# Elucidating the endogenous synovial fluid proteome and peptidome of inflammatory arthritis using label-free mass spectrometry

**DOI:** 10.1186/s12014-019-9243-3

**Published:** 2019-05-30

**Authors:** Shalini M. Mahendran, Edward C. Keystone, Roman J. Krawetz, Kun Liang, Eleftherios P. Diamandis, Vinod Chandran

**Affiliations:** 1 Department of Laboratory Medicine and Pathobiology, University of Toronto, Toronto, ON Canada; 2 Department of Pathology and Laboratory Medicine, Mount Sinai Hospital, Toronto, ON Canada; 3 Department of Rheumatology, Mount Sinai Hospital, Toronto, ON Canada; 4 McCaig Institute for Bone and Joint Health, University of Calgary, Calgary, AB Canada; 5 Department of Surgery, University of Calgary, Calgary, AB Canada; 6 Department of Anatomy and Cell Biology, University of Calgary, Calgary, AB Canada; 7 Department of Statistics and Actuarial Science, University of Waterloo, Waterloo, ON Canada; 8 Department of Clinical Biochemistry, University Health Network, Toronto, ON Canada; 9 Centre for Prognosis Studies in Rheumatic Diseases, Krembil Research Institute, Toronto Western Hospital, University Health Network, Toronto, ON Canada; 10 Division of Rheumatology, Department of Medicine, University of Toronto, Toronto, ON Canada; 11 Institute of Medical Science, University of Toronto, Toronto, ON Canada

**Keywords:** Proteomics, Peptidomics, Biomarkers, Synovial fluid, Mass spectrometry, Inflammatory arthritis, Rheumatoid arthritis, Psoriatic arthritis

## Abstract

**Background:**

Inflammatory arthritis (IA) is an immunological disorder in which loss of immune tolerance to endogenous self-antigens perpetuates synovitis and eventual destruction of the underlying cartilage and bone. Pathological changes in the joint are expected to be represented by synovial fluid (SF) proteins and peptides. In the present study, a mass spectrometry-based approach was utilized for the identification of key protein and peptide mediators of IA.

**Methods:**

Age-matched SF samples from 10 rheumatoid arthritis patients, 10 psoriatic arthritis patients and 10 cadaveric controls were subjected to an integrated proteomic and peptidomic protocol using liquid chromatography tandem mass spectrometry. Significant differentially abundant proteins and peptides were identified between cohorts according to the results of a Mann–Whitney U test coupled to the Benjamini–Hochberg correction for multiple hypothesis testing. Fold change ratios were computed for each protein and peptide according to their log-transformed extracted ion current. Pathway analysis and antimicrobial peptide (AMP) prediction were conducted to clarify the pathophysiological relevance of identified proteins and peptides to IA.

**Results:**

We determined that 144 proteins showed significant differential abundance between the IA and control SF proteomes, of which 11 protein candidates were selected for future follow-up studies. Similar analyses applied to our peptidomic data identified 15 peptide sequences, originating from 4 protein precursors, to have significant differential abundance in IA compared to the control SF peptidome. Pathway enrichment analysis of the IA SF peptidome along with AMP prediction suggests a possible mechanistic role of microbes in eliciting an immune response which drives the development of IA.

**Conclusions:**

The discovery-phase data generated herein has provided a basis for the identification of candidates with the greatest potential to serve as novel serum biomarkers specific to inflammatory arthritides. Moreover, these findings facilitate the understanding of possible disease mechanisms specific to each subtype.

**Electronic supplementary material:**

The online version of this article (10.1186/s12014-019-9243-3) contains supplementary material, which is available to authorized users.

## Introduction

Inflammatory arthritis (IA) is characterized by synovial hyperplasia leading to degradation of adjacent articular cartilage and bone [1]. The term encompasses several forms of inflammatory joint diseases that when taken together, have an annual incidence ranging from 115 to 271 per 100,000 adults [2]. IA is a multifactorial disease driven by the complex interplay of both genetics and the environment. Rheumatoid arthritis (RA), the most common and potentially destructive IA, has a well-established association with class II major histocompatibility complex (MHC) alleles while the spondyloarthritides, such as psoriatic arthritis (PsA), are more frequently associated with class I MHC alleles [3]. Susceptibility to IA increases when genetic predisposition is complemented by environmental risk factors such as smoking, obesity and more recently, microbial infection and intestinal dysbiosis [4–6]. The exact etiology of IA is still poorly understood with studies aimed at delineating the molecular pathways driving loss of immunological tolerance to the body’s self-antigens. Alterations to the adaptive and innate immune system perpetuate systemic inflammation and lead to an elevated risk of developing comorbid conditions such as cardiovascular disease, metabolic syndrome, diabetes and depression [7, 8]. Naturally, there is a compelling need to identify markers of aberrant immune pathways relevant to IA which may advance current insights into the molecular mechanisms of the disease and serve as clinical markers for disease monitoring and treatment responses.

The rise in high-throughput technologies, such as next-generation gene sequencing and mass spectrometry (MS), facilitate the discovery of key modulators of disease. Specifically, MS-based approaches provide an essential analytical platform for the identification, quantification and characterization of candidate biomarkers. Biomarkers may come in the form of a molecular signature, a clinical feature or even as an imaging parameter. Molecular biomarkers may be further subtyped into the domains of genomics, transcriptomics, proteomics, metabolomics or peptidomics. Due to the importance of proteins in pathophysiological processes, there is increased interest in resolving the proteomic profile of biospecimens related to IA. Similarly, peptides play a seminal role in mediating physiological functions by serving as neurotransmitters, hormones, antibiotics and immune regulators [9]. During IA, joint pain and inflammation are driven by aberrant proteolysis resulting in the production of inflammatory peptides and the destruction of joint cartilage and bone. Synovial fluid (SF), a proximal fluid which bathes the intrinsic joint structures, is an important reservoir of putative protein and peptide biomarkers whose abundance levels fluctuate in response to pathological changes due to disease [10].

In the current study, we performed MS-based proteomic and peptidomic analyses of SF from RA and PsA patients to identify and quantify significant proteins and peptides related to the aetiopathogenesis of IA. Differential abundance analyses highlighted the capacity for dysregulated SF proteins and peptides to reflect disease activity while pathway analysis and antimicrobial peptide (AMP) prediction alluded to a larger role of microbes in the initiation and progression of IA. These findings provide the means for discovering novel candidates to serve as possible biomarkers of IA while simultaneously, highlighting possible mechanistic networks responsible for the disease progression of RA and PsA.

## Materials and methods

### Patients and SF collection

Research ethics board approval was received for the study from the University Health Network, Mount Sinai Hospital and the University of Calgary. Informed consent was obtained from all patients.

SF samples for the study were obtained, retrospectively, from 10 cases with RA, 10 cases with PsA and 10 cadaveric controls. RA patients were classified according to the 1987 American College of Rheumatology (ACR) classification criteria [11]. PsA patients satisfied the Classification Criteria for Psoriatic Arthritis (CASPAR) [12].

Cadaveric control SF were obtained from joints through the Southern Alberta Tissue Donation Program. Inclusion criteria consisted of an age of 18 years or older with no medical history of arthritis, joint injury or joint surgery (including visual inspection of cartilage surfaces during recovery), no prescription anti-inflammatory medications and availability within 4 h of death. Exclusion criteria for all disease cohorts included patients receiving therapeutic biological drugs and the presence of other causes of inflammation (e.g. infection and/or crystal disease) or co-morbidities (e.g. cancer).

### SF sample preparation

IA SF samples (both RA and PsA) were obtained through needle aspiration of knee joints and kept on ice. Samples were transferred to centrifuge tubes and spun at 160 RCF for 10 min at 4 °C. The supernatant was transferred to a sterile 1.5 mL centrifuge tube and spun at 2000 RCF for another 10 min at 4 °C. Samples were immediately stored at − 80 °C until further processing. SF samples from cadavers were collected without the use of lavage. Samples were centrifuged at 3000 RCF for 15 min and stored at − 80 °C until further processing.

At the time of analysis, samples were blinded, randomized, thawed on ice and their respective total protein concentrations were measured with a Pierce Coomassie (Bradford) total protein assay.

### SF sample preparation for proteomic analysis

For proteomic investigations, SF samples were first adjusted to 300 µg total protein in 50 mM ammonium bicarbonate (ABC). Protein concentration was conducted using Amicon Ultra-0.5 centrifugal filter units (10 kDa molecular weight cut-off; MilliporeSigma) which were pre-equilibrated with 400 uL of 50 mM ABC. Samples were loaded and spun at 10,000 RPM for 35 min at 4 °C and transferred to a new tube by spinning upside at 5000 RPM for 2 min.

Concentrates were collected and brought to a total volume of 100 µL with 50 mM ABC. Proteins were denatured with powdered urea to a final concentration of 8 M. Dithiothreitol (DTT) (Sigma-Aldrich) was added to each concentrate sample to a final concentration of 5 mM and incubated at 60 °C for 45 min. This was followed by alkylation with 15 mM iodoacetamide (IAM) (Sigma-Aldrich) at room temperature in the dark for 45 min. Samples were diluted fivefold with 50 mM ABC to prevent inhibition of trypsin activity by high concentrations of urea. Concentrate samples were digested with trypsin (Sigma-Aldrich) in a 1:50 (trypsin to total protein) ratio for 20 h at 37 °C and then dropwise acidified to a pH of 2 with formic acid (FA) to inhibit trypsin activity. Samples were reduced to 300 µL via speed vacuum concentration and stored at − 20 °C until subjected to liquid chromatography-tandem mass spectrometry (LC–MS/MS).

### SF sample preparation for peptidomics analysis

Peptides were isolated based on a protocol described by Kamphorst et al. [13]. Fifty microliters of SF were diluted in 235 µL of 50 mM ABC and 15 µL dimethyl sulfoxide (DMSO) for peptidomic analysis. Peptide concentration was conducted using Amicon Ultra-0.5 centrifugal filter units (10 kDa MWCO; MilliporeSigma) which were pre-equilibrated with 250 µL of 50 mM ABC. SF samples were spun at 10 000 RPM for 60 min at 4 °C then washed with 100 µL of 50 mM of ABC and spun for another 10 min. Filtrates were acidified with 5 µL of FA.

Peptides were desalted using one hydrophilic-lipophilic-balanced reverse-phase cartridge per sample (Oasis HLB). Each cartridge [1 mL (30 mg); Waters cat# WAT094225] was first pre-equilibrated with 1 mL of 90% acetonitrile (ACN), 0.1% FA and 0.02% trifluoroacetic acid (TFA) and then washed with 3 mL of buffer A (5% ACN, 0.1% FA, 0.02% TFA). The SF sample was then passed through the cartridge and washed a second time with 3 mL of buffer A. Peptides were eluted with 700 µL of 60% ACN, 0.1% FA and 0.02% TFA and each eluate was reduced to a volume of less than 300 µL and stored at − 20 °C until subjected to LC–MS/MS.

### LC–MS/MS

Processed samples were desalted using C-18 OMIX Pipette Tips (Agilent Technologies, USA) and eluted in 3 µL of MS buffer B (65% ACN, 0.1% FA in H_2_O). The eluates were then diluted with 57 µL of MS buffer A (0.1% FA in H_2_O) and 28 µL were injected onto a 2 cm C18 trap column, packed with Varian Pursuit (5 µm C18), with an 8 µm tip (New Objective). The LC setup was coupled online to a Q Exactive (Thermo Fisher Scientific, USA) mass spectrometer with a nanoelectrospray ionization source (Proxeon Biosystems). Samples for direct proteomic analysis as well as samples for direct peptidomics analysis underwent a 60-min linear gradient using MS buffer A and MS buffer B. Eluted peptides were subjected to tandem mass spectrometry in positive ion mode. Data acquisition was conducted via Thermo XCalibur v.3.0.63 (Thermo Fisher Scientific, USA).

### Protein identification and quantification

The resulting proteomic and peptidomic raw data files were uploaded into MaxQuant v.1.5.2.8 (www.coxdocs.org) [14] with the integrated Andromeda search engine. MS and MS/MS spectra were searched against a reverted version of the SwissProt human protein database (version July 2017) for protein identification and a randomized version of the SwissProt human protein database for peptide identification. Search parameters for proteomic analysis included carbamidomethylation of cysteines as a fixed modification and oxidized methionine and N-terminal acetylation as variable modifications. Trypsin was the chosen digestion enzyme and a maximum of two missed cleavages were allowed. Search parameters for peptidomic analysis included oxidized methionine and oxidized proline as variable modifications. An unspecific enzyme search was the chosen digestion method. Both proteomic and peptidomic data were initially searched against a smaller “human first search” database with a peptide tolerance of 20 ppm for mass recalibration. The main search was performed using the Swissprot human protein database (version July 2017) with a peptide tolerance of 4.5 ppm. Data was analyzed using label-free quantification (LFQ) with a minimum ratio count of 1 and the “Match between runs” interval set to 2 min. The peptide-spectrum match and protein false discovery rate were set to 1%.

### Bioinformatic analyses

Pathway analysis of dysregulated proteins identified by LC–MS/MS was conducted using the functional-analysis tool Ingenuity Pathway Analysis (IPA; http://www.ingenuity.com) [15]. To determine the specificity of identified proteins at the tissue and biological fluid level, proteomic datasets were searched against ProteomicsDB (http://www.ProteomicsDB.org), a web-based database of mass spectrometry-generated proteomics data [16]. Pathway analysis of SF peptides was conducted through the Database for Annotation, Visualization and Integrated Discovery (DAVID) 6.8 with reference to the Kyoto Encyclopedia of Genes and Genomes (KEGG) [17]. Annotations with q-values of less than 0.05 were considered statistically significant. Identification of known AMPs in the SF peptidome was determined by comparison with experimentally validated human AMPs taken from the Collection of Anti-Microbial Peptides (CAMP_R3_) (http://www.camp.bicnirrh.res.in/) database [18]. AMP prediction of the identified peptides was performed using the support vector machine (SVM) learning algorithm developed for the CAMP_R3_ database. Peptides with an SVM score of 0.8 or higher were predicted to be antimicrobial.

### Statistical analyses

Statistical analyses and data visualizations were completed with R (R Foundation for Statistical Computing). A linear model was fitted to examine the effects of age and sex on the protein and peptide expression data using the LIMMA package in R [19]. Due to the nature of data generated by LC–MS/MS, protein quantification is often skewed and imposes limits on statistical inference. To circumvent assumptions of normality, the Mann–Whitney U test coupled to the Benjamini–Hochberg correction to control for multiple hypothesis testing was performed for comparisons between two independent groups. Adjusted *p* values of less than 0.05 were considered statistically significant. Differential abundance of proteins and peptides were computed with the myTAI package in R, generating a ratio of log-transformed extracted ion currents in one group against the second group, considered to be the reference group [20]. A volcano plot was used to visualize the results of the Mann–Whitney U test.

## Results

### Clinical characteristics of recruited patients

Demographics, disease characteristics and concomitant therapies of recruited patients are summarized in Table 1.

**Table 1:** Demographics, disease characteristics and concomitant therapies of subjects (RA, PsA and control) from whom the samples were obtained

Characteristics	RA (n = 10)	PsA (n = 10)	Control (n = 10)
Females (%)	80	10	50
Age (years)	55 ± 13.5	43 ± 12.0	61 ± 9.9
Total actively inflamed joints	NA	1.9 ± 1.7	–
Swollen joint count	NA	1.2 ± 1.2	–
Tender joint count	NA	1.3 ± 1.5	–
With dactylitis (%)	–	40	–
With enthesitis (%)	–	0	–
Total clinically damaged joints	NA	7.8 ± 11.3	–
On NSAIDs (%)	10%	60%	–
On DMARDs (%)	50%	40%	–

Values are reported as the mean ± standard deviation unless otherwise indicated

*NA* not available

### Holistic protein and peptide mining

Collectively, 389 unique proteins were identified across all IA SF proteomic samples. When assessing each cohort individually, 377 unique proteins were identified in RA patient samples, 369 unique proteins were identified in PsA patient samples and 399 proteins were identified in control patient samples. A review of the overlap between proteomes of each cohort revealed 347 proteins to be common to all three patient groups.

A total of 226 unique peptide sequences were identified across all IA SF samples originating from a total of 48 unique proteins. Inter-cohort comparisons identified 184 unique peptides in RA patient samples, 175 unique peptides in PsA patient samples and 192 unique peptides in control patient samples. Comparisons between the SF peptidomes of arthritic and control conditions revealed 95 peptides to be common to all three groups.

**Fig. 1:**
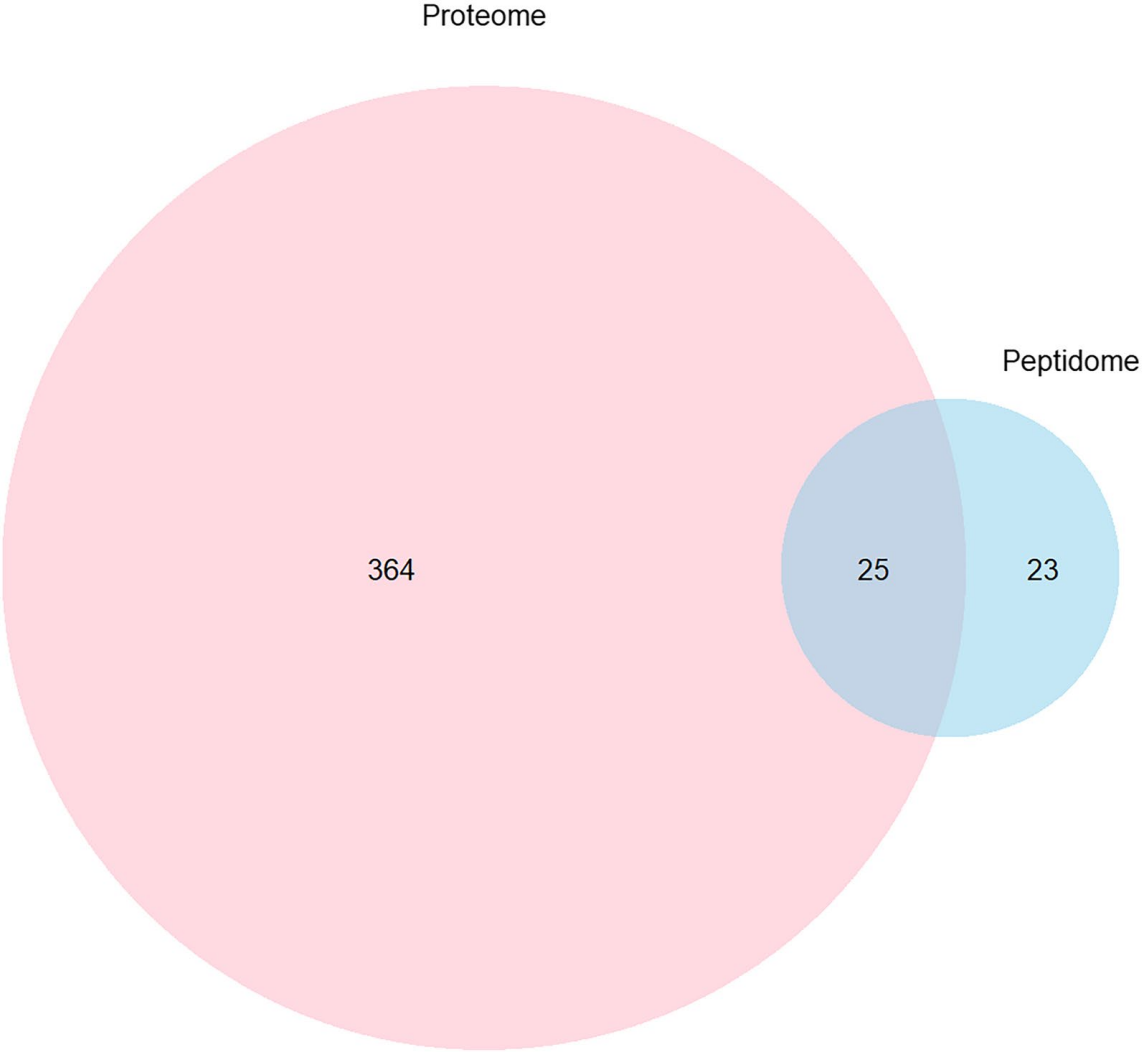
Venn diagram of proteins identified in the IA SF proteome and peptidome. The total number of proteins identified was 412, with 364 proteins detected in the proteome, 23 proteins detected in the peptidome and 25 proteins detected in both

Next, we investigated the overlap between the proteins identified through our peptidomic approach and those identified through our proteomic approach by comparing the IA-associated proteins originating from both experiments. Of the 48 precursor proteins from our peptidomic study, 25 proteins were also found in the IA SF proteome (Fig. 1). Taken together, they have yielded the combined identification of 412 proteins in IA SF. A complete list of identified proteins and peptides are reported in Additional file 1: Tables S1, S2 and S3.

### Dysregulated proteins in IA SF

**Fig. 2:**
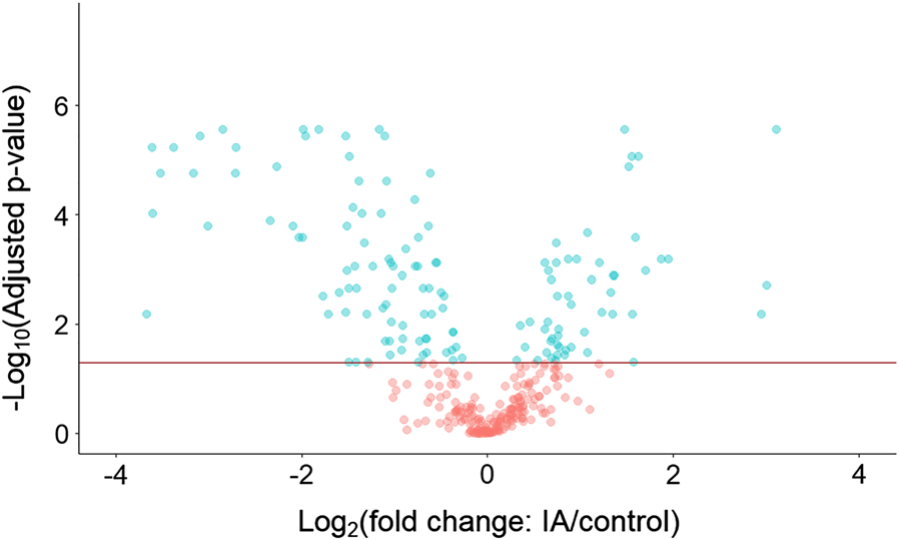
Volcano plot of significantly differentially abundant proteins identified in the IA SF proteome relative to control SF. A total of 144 proteins, highlighted in blue and found above the y-intercept of − log_10_(0.05), were determined to have statistically significant differential abundance in IA SF

Differential abundance analyses were conducted to detect dysregulated proteins in the SF of: (1) IA compared to control and (2) RA compared to PsA. Using non-parametric statistical tests, 144 proteins were determined to have statistically significant differential abundance in IA SF with 54 proteins showing significant upregulation and 90 proteins showing significant downregulation (Fig. 2). When comparing RA and PsA proteomes, no proteins showed significant differences in abundance after correcting for multiple hypothesis testing. However, with respect to an unadjusted *p* value, 22 proteins were differentially abundant between the two groups with 13 proteins demonstrating significant upregulation in RA relative to PsA and 9 proteins showing significant upregulation in PsA relative to RA. Significantly dysregulated proteins in IA compared to control and significantly dysregulated proteins in RA compared to PsA are summarized in Additional file 1: Tables S4 and S5, respectively.

Dysregulated functional pathways likely to be associated with the significantly upregulated and downregulated proteins of IA SF were detected with IPA. Core analyses determined the top 5 canonical pathways associated with upregulated proteins to be: (1) LXR/RXR activation, (2) FXR/RXR activation, (3) acute phase response signaling, (4) atherosclerosis signaling and (5) IL-12 signaling and production in macrophages, several of which have been previously associated with IA. Details regarding the top diseases and disorders as well as molecular and cellular functions associated with both groups of dysregulated proteins can be found in Additional file 1: Table S6. As the data suggests, upregulated proteins show more distinct relations to inflammatory and immunological processes while downregulated proteins demonstrate stronger relations to metabolic processes. Ultimately, to identify the strongest candidate biomarkers to be validated in IA patient serum, we focused on upregulated proteins in the SF.

Tissue and fluid specificity of upregulated proteins were used to narrow down the list of candidates deemed likely to be associated with IA, RA and PsA. We concentrated on proteins which displayed strong abundance in SF, bone, bone marrow or immune regulatory cells according to ProteomicsDB. Immunoglobulins were excluded from further analysis. The resulting list of upregulated proteins compared to the reference group consisted of 38 IA-specific, 8 RA-specific and 9 PsA-specific unique protein candidates. High abundance proteins in serum, as identified according to the literature [21, 22], were excluded due to the likelihood that they were serum contaminants at the time of joint aspiration. Moreover, this ensured protein candidates were unlikely to be overexpressed in the serum of non-diseased patients. Following this filtering step, the final list of candidate biomarkers consisted of 5, 4 and 2 upregulated proteins which we deemed likely to be associated with IA, RA and PsA, respectively (Table 2).

**Table 2:** Fold change ratios of selected upregulated protein candidate biomarkers of IA, RA and PsA

Condition	Protein name	Gene name	Fold change ratio	*p* value	Adjusted *p* value
IA	Stromelysin-1	MMP3	24.01	2.00E−06	2.46E−05
	Defensin alpha 3	DEFA3	2.98	2.16E−02	4.97E−02
	Alpha-ketoglutarate-dependent dioxygenase	FTO	1.70	9.62E−03	2.62E−02
	WASH complex subunit	FAM21C	1.57	2.05E−04	1.06E−03
	T-box transcription factor	TBX3	1.54	1.26E−04	7.50E−04
RA	Low affinity immunoglobulin gamma Fc region receptor	FCGR3A	1.42	1.15E−02	4.06E−01
	Coagulation factor XII	F12	1.40	4.33E−02	5.98E−01
	SPARC-like protein 1	SPARCL1	1.36	1.47E−02	4.06E−01
	Rab GDP dissociation inhibitor beta	GDI2	1.25	1.47E−02	4.06E−01
PsA	Periostin	POSTN	1.72	2.32E−02	4.54E−01
	Phosphoglycerate kinase 1	PGK1	1.29	1.47E−02	4.06E−01

### Dysregulated peptides in IA SF

Differential abundance analyses were conducted to detect strongly dysregulated peptides in the SF of: (1) IA compared to control and (2) RA compared to PsA. For both comparisons, no peptides showed statistically significant differences in abundance after correcting for multiple hypothesis testing, with the exception of the peptide sequence DSGEGDFLAEGGGV when comparing IA to the control. Alternatively, with respect to the unadjusted *p* value, 11 peptides were determined to be significantly differentially abundant in IA SF with 10 peptides showing significant upregulation and 1 peptide showing significant downregulation (Table 3). A complete list of dysregulated peptides in IA compared to control and dysregulated peptides in RA compared to PsA are summarized in Additional file 1: Tables S7 and S8, respectively.

**Table 3:** Significant differentially abundant peptides between IA and control SF, as identified by LC-MS/MS

Upregulated peptides in IA					Downregulated peptides in IA				
Gene name	Sequence	Fold change	*p* value	Adjusted *p* value	Gene name	Sequence	Fold change	*p* value	Adjusted *p* value
FGA	DSGEGDFLAEGGGV	15.33	1.62E−04	1.21E−02	FGB	EEAPSLRPAPPP	0.10	7.18E−03	7.69E−02
COL1A1	GPPGPPGPPGPPG	3.88	1.62E−03	6.08E−02					
FGA	DFLAEGGGVR	6.82	3.26E−03	7.69E−02					
FGA	EGDFLAEGGGVR	4.29	4.51E−03	7.69E−02					
FGA	GEGDFLAEGGGVR	6.16	5.29E−03	7.69E−02					
FGA	GDFLAEGGGVR	4.26	6.17E−03	7.69E−02					
CCSER2	YMWDEEGLEPI	8.99	1.46E−02	1.37E−01					
FGA	FLAEGGGVR	6.30	1.90E−02	1.58E−01					
FGA	SGEGDFLAEGGGVR	4.62	2.76E−02	2.07E−01					
FGA	EGDFLAEGGGV	3.46	3.11E−02	2.12E−01					

When comparing RA and PsA peptidomes, 5 peptides showed differential abundance between the two groups with all 5 peptides demonstrating significant upregulation in PsA SF relative to RA SF (Table 4).

**Table 4:** Significant differentially abundant peptides between RA and PsA SF, as identified by LC-MS/MS

Gene name	Sequence	Fold change PsA versus RA	*p* value	Adjusted *p* value
FGB	EEAPSLRPAPPPISGGGY	6.12	2.09E−03	1.57E−01
FGA	ALTDMPQM	6.06	5.20E−03	1.95E−01
FGA	VPDLVPGNF	4.37	1.15E−02	2.87E−01
FGA	ADSGEGDFLAEGGGVR	3.08	2.32E−02	4.36E−01
COL1A1	RPGEVGPPGPPGP	2.83	4.33E−02	5.91E−01

### Pathway enrichment analysis of the SF peptidome

**Fig. 3:**
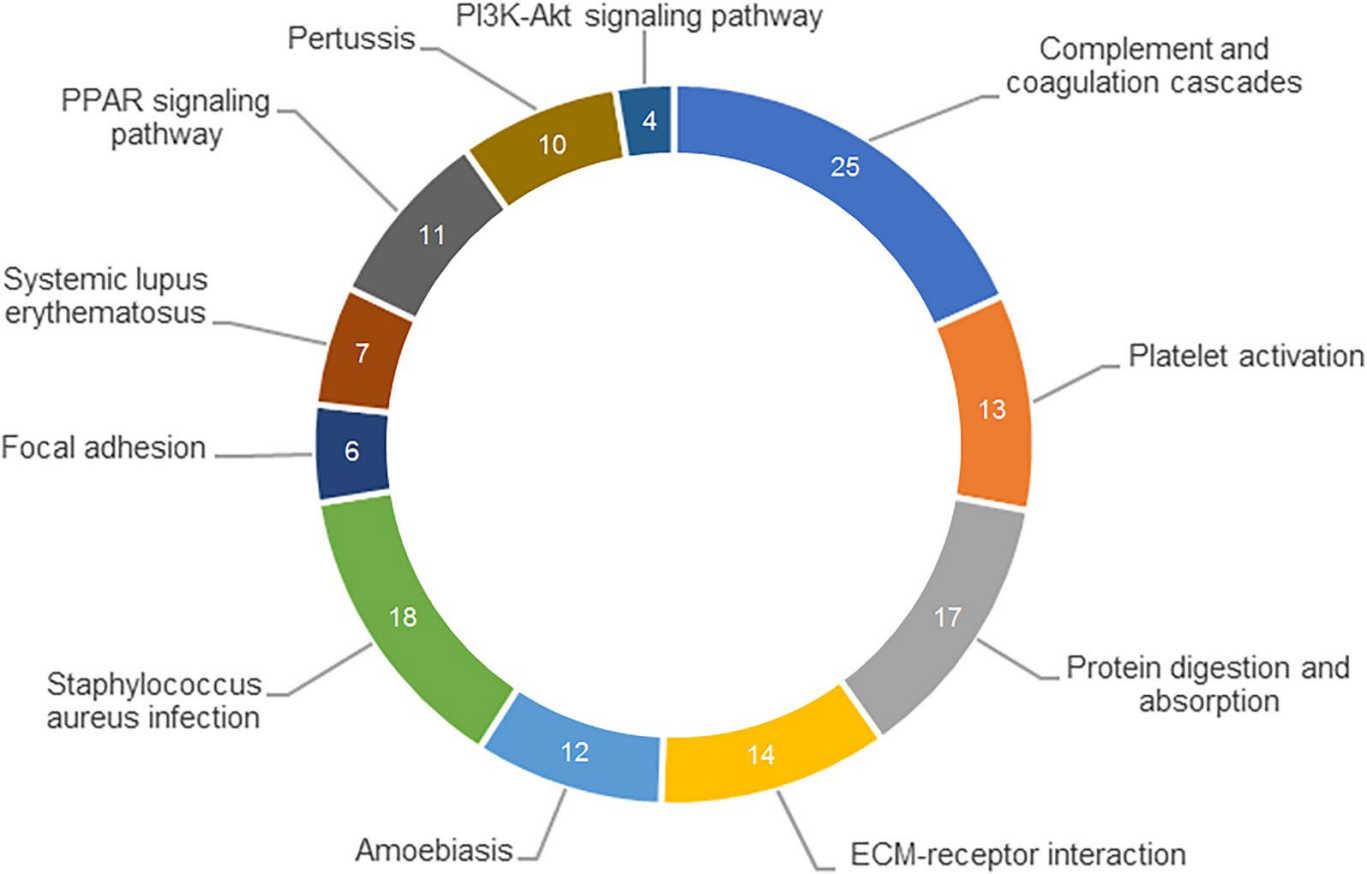
KEGG enrichment analysis of precursor proteins in the IA SF peptidome. Top significantly enriched pathways included complement and coagulation cascades, *Staphylococcus aureus* infection, protein digestion and absorption and ECM-receptor interaction

KEGG analysis revealed significantly enriched pathways (fold enrichment in brackets) related to the IA SF peptidome. Figure 3 illustrates the top KEGG pathways among which complement and coagulation cascades [23], *Staphylococcus aureus* infection [18], protein digestion and absorption [17] and extracellular matrix (ECM)-receptor interaction [14] were significantly enriched.

### Antimicrobial peptides in IA SF

Accumulating evidence suggests a crucial role of intestinal resident flora in chronic activation of innate and adaptive immune responses leading to inflammatory disorders. Microorganisms residing in the intestine play an important role in maintaining systemic homeostasis through the delicate balance of the immune system response. Perturbations in the composition of the intestinal microbiota have been shown to elicit inappropriate immune cell activation leading to an inflammatory cascade and eventually, clinical disease [24]. Specifically, perturbations of the gut epithelial cell layer and/or increased exposure to microbial metabolites may be primary triggers of an inflammatory cascade resulting in joint pathology [25]. Protective mechanisms, such as the expression of AMPs, have naturally developed to oppose microbial dysbiosis. AMPs are fundamental effectors of the innate immune response with a broad spectrum of microbicidal activity. Under inflammatory conditions, the synovial membrane has demonstrated an altered pattern of expression of AMPs relative to healthy controls and suggests a valuable role of these proteins in the differential diagnosis of inflammatory joint disease [23].

Putative AMPs in the SF peptidome of IA were predicted with the assistance of a SVM learning algorithm (Additional file 1: Table S9). Overall, 26 peptide sequences originating from 8 proteins (complement C4-A, fibrinogen beta chain, fibrinogen alpha chain, annexin A1, collagen type III alpha 1 chain, collagen type I alpha 1 chain, gliomedin and EMI domain-containing protein (1) were predicted to have antimicrobial activity with an SVM score of 0.8 or higher (Table 5).

**Table 5:** Complete list of all predicted AMPs in IA SF

Protein ID	Gene name	AA before	Sequence	AA after	AMP probability
P0C0L4-2	C4A	K	DDPDAPLQP	V	1
P02675	FGB	N	DNEEGFFS	A	1
P02675	FGB	N	DNEEGFFSA	R	1
P04083	ANXA1	I	ENEEQEYVQT	V	1
P02675	FGB	V	NDNEEGFF	S	1
P02675	FGB	V	NDNEEGFFS	A	1
P02675	FGB	V	NDNEEGFFSA	R	0.998
P04083	ANXA1	E	NEEQEYVQTV	K	0.994
P02675	FGB	A	PPPISGGGY	R	0.988
P02671-2	FGA	A	DSGEGDFL	A	0.981
P0C0L4-2	C4A	K	DDPDAPLQPV	T	0.979
P02671-2	FGA	F	SPMLGEFV	S	0.946
P02671-2	FGA	G	DSTFESKSY	K	0.939
P02671-2	FGA	A	DSGEGDFLA	E	0.923
P02671-2	FGA	F	DTASTGKTFPGFFSPM	L	0.914
P02675	FGB	V	NDNEEGFFSAR	G	0.911
P02671-2	FGA	F	DTASTGKTFP	G	0.909
P02452	COL1A1	V	GPPGPPGPPGPPGPP	S	0.868
P02452	COL1A1	P	GPPGPPGPPGPP	G	0.862
Q96A84-2	EMID1	P	PGPPGPPGPPGPPAP	V	0.851
P02452	COL1A1	P	GPPGPPGPPGPPG	L	0.842
Q6ZMI3-2	GLDN	Q	GPPGPPGPPGPPGPPG	P	0.842
P02452	COL1A1	P	GPPGPPGP	P	0.834
P02452	COL1A1	P	VGPPGPPGPPGPPGPP	S	0.833
P02452	COL1A1	P	VGPPGPPGPPGPP	G	0.824
P02461	COL3A1	P	GPAGPPGPPGPPG	T	0.818

## Discussion

In the current study, a comparative MS-based approach coupled to statistical and bioinformatics analyses was performed on IA SF relative to control SF, and RA SF relative to PsA SF, to detect notable differences in both the proteomic and peptidomic data. Studies using an MS-based approach to evaluate the proteome of similar inflammatory diseases, including psoriasis [26], systemic lupus erythematosus [27], and ankylosing spondylitis [28], corroborate the robustness of such analytical methodologies. The investigation of a proximal joint fluid, such as SF, was preferred since its protein and peptide expression patterns are expected to be reflective of the pathophysiological state of the joint. As such, elucidating the SF proteome and peptidome during the progression of IA can provide novel insights into molecular drivers of the disease.

The molecular pathways involved in the pathogenesis of IA are also overrepresented in the current study based on functional network analysis of IA SF proteins and peptides. Prominent mechanisms related to the identification of upregulated proteins include: (1) acute phase response signaling, (2) antimicrobial response, (3) inflammatory response, (4) IL-12 signaling and production in macrophages and (5) cell-to-cell signaling and interaction. Similarly, interaction networks were established through pathway enrichment analysis of IA SF peptides. Of interest was the enrichment of *Staphylococcus aureus* infection. As previously highlighted, correlative studies are beginning to recognize a fundamental interplay between the microbiome and immune system response in the etiology of IA [29, 30]. Although the role of *S. aureus* in the progression of IA has yet to be clarified, the enrichment of this pathway, as reflected by the peptides identified in our study, reinforces this developing hypothesis.

Overall, our analyses resulted in the identification of 144 differentially expressed proteins in the IA SF proteome. Comparison of RA SF to PsA SF identified 22 differentially expressed proteins. Since we are interested in identifying putative markers which can be further validated in patient serum, we decided to focus solely on upregulated proteins in each arthritic condition. High-potential candidate biomarkers were selected on the basis of several molecular features including: differential abundance, fluid and tissue specificity, immunoglobulin status and abundance in the plasma proteome. Our list of dysregulated proteins in IA was reduced to a total of 5 promising protein candidates representative of intrinsic joint structures including the articular cartilage, synovial membrane and synoviocytes. The re-discovery of several upregulated proteins which have been previously implicated in IA, such as CD5 molecule-like (CD5L), matrix metalloproteinase (MMP)-3, defensin alpha 3 (DEFA3), S100 calcium-binding protein (S100) A8, and A9, provided an internal validation of our analytical proteomic approach [31, 32]. The application of similar, stringent filtering criteria on protein candidates of RA and PsA resulted in 4 RA-specific and 2 PsA-specific promising protein candidates.

Our analytical approach also yielded the discovery of novel putative biomarkers which, to our knowledge, have yet to be described in the context of IA. This includes the identification of alpha-ketoglutarate-dependent dioxygenase (FTO), family with sequence similarity 21 member C (FAM21C; more commonly known as WASH complex subunit 2C, WASHC2C) and T-box transcription factor (TBX3). Of these candidates, only TBX3 has been previously observed in IA at the genetic level [33]. A genome-wide association study (GWAS) identified the single nucleotide polymorphism (SNP), rs12579024, located nearest the TBX3 gene, to be strongly associated with RA in a Korean population (*p* value < 0.0001). The functional roles of TBX3 have, thus far, been primarily described in relation to the morphogenesis of limbs and organs [34] as well as oncogenic processes [35]. A recent study by Willmer et al. [36] attempted to delineate the molecular mechanisms driven by TBX3 and identified cyclin-dependent kinase inhibitor p21^WAF1^ (p21), a key mediator of cell cycle arrest, to be a primary repressed target of TBX3. Interestingly, p21 has also been implicated in the regulation of proinflammatory cytokines and MMP production in synovial fibroblasts, both of which greatly promote inflammation and joint destruction during the pathogenesis of RA [37]. Isolated RA synovial fibroblasts have shown reduced expression of p21 relative to osteoarthritis (OA) synovial fibroblasts and adenovirus-mediated delivery of p21 suppresses the spontaneous production of IL-6 and MMP1 in RA synovial fibroblasts. In support of this, p21^−/−^ mice maintain an enhanced experimental IA with markedly increased numbers of macrophages and articular destruction [38]. This phenotype is resolved, however, with the administration of a p21-peptide mimetic. When taken with our own findings, it is conceivable that the upregulation of TBX3 in the synovial joint may lead to reduced p21 expression in synovial fibroblasts and promotes the proinflammatory state distinctive of IA pathogenesis. These findings corroborate with our hypothesis that delineating the IA proteome may highlight underlying mechanisms related to the progression of inflammatory arthritic disease and serve as novel targets for screening and therapeutic purposes.

Comparisons of RA and PsA revealed high-priority protein candidates specific to each disease. In RA SF, coagulation factor XII, SPARC-like protein 1, Rab GDP dissociation inhibitor beta and immunoglobulin gamma Fc region receptor III-A (FCGR3A) were notably upregulated; of which, activating FCGR3A has demonstrated important roles in sustaining the inflammatory response through the secretion of cytokines and proteases from the immune cell on which it is expressed [39]. Likewise, allelic studies have demonstrated SNPs that may serve as susceptibility markers for RA [40]. Taken together, the therapeutic targeting of FCGR3A may facilitate future management of RA.

Of the two PsA-specific protein candidates we identified, periostin (POSTN) has been previously investigated in our studies of the PsA tissue proteome as a potential serum marker of PsA [41]. Although serum validation of POSTN did not reveal statistically significant differences between PsA and control serum, its elevated levels in both PsA lesional skin as well as SF alludes to an important role of the protein in the pathobiology of PsA and may serve as part of a panel of biomarkers to differentiate between the onset of PsA and RA.

Differential abundance analyses of peptide sequences identified 11 peptides to be significantly dysregulated in IA SF compared to the control group. Upregulated peptide sequences were primarily derived from FGA while single sequences originated from collagen type I alpha 1 (COL1A1) and coiled-coil serine rich protein 2 (CCSER2). All significant FGA-derived peptide fragments were representative of the 16-amino acid residue (ADSGEGDFLAEGGGVR) of fibrinopeptide A (FpA) located at the NH_2_-terminal end of FGA. The lack of detection of the full-length FpA peptide sequence in IA SF can be rationalized by the peptide’s short half-life of 3–5 min in the blood plasma [42]. FpA is a cleavage product of thrombin-induced conversion of fibrinogen into a fibrin clot. Fibrin deposition in the SF or on the synovial membrane is a consistent feature of IA and is believed to perpetuate inflammation and joint tissue destruction through synovial cell activation [43, 44]. Liu et al. demonstrated that stimulation of synovial fibroblasts with fibrin(ogen) resulted in the upregulated expression of IL-8 and intercellular adhesion molecule 1 (ICAM-1) for the recruitment and retention, respectively, of lymphocytes within the arthritic joint [43]. Elevated abundance of FGA and FpA in serum has been observed in patients with inflammation-associated diseases including systemic lupus erythematosus, Crohn’s disease, ischemic heart disease and gastric cancer [45–48]. These findings highlight the non-specific indication of inflammation by FpA and its associated peptide fragments, and advocates for its utility as a sensitivity index of disease activity in patients with IA. Moreover, targeting FGA in the synovial joint may be a necessary therapeutic intervention to modulate the inflammatory response. Comparisons of peptide abundance between RA and PsA identified FGA and FGB-related peptide sequences to be consistently downregulated in RA relative to PsA. Although this may be indicative of a discriminatory ability for FGA and FGB peptide fragments to differentiate between the onset of RA and PsA, this outcome does not corroborate with the finding that RA patients are at a greater increased risk of venous thromboembolism relative to PsA patients [49]. Targeted quantification in a second set of SF samples is necessary to verify this finding.

The advent of high-throughput microbial DNA sequencing has marked a renewed interest in the complex interplay of the intestinal microbiome and inflammatory diseases. Studies suggest that the induction of autoimmunity is closely linked to intestinal dysbiosis and leads to distal synovitis and joint pathology [50]. There exist several protective mechanisms to prevent changes in the gut microbiota including the physicochemical barrier of antimicrobial proteins and peptides. AMPs are a collective of naturally-occurring, cationic peptides released by lymphocytes of the innate immune system. Of the 26 peptides predicted to have antimicrobial activity, 13 of them originated from FGA or FGB precursor proteins. Despite the pro-inflammatory impression associated with the accumulation of FGA and FGB in the SF, their presence may be critical to the activation of microbicidal activity. Soluble fibrinogen and fibrin matrices have demonstrated antimicrobial host defense through their ability to physically entrap bacteria in addition to the recruitment and engagement of host immune cells which in turn, facilitate the removal of invading pathogens [51]. Taken together, the deposition of fibrin during the progression of IA may initially serve the favourable purpose of limiting bacterial infection through the activation of antimicrobial host defense mechanisms. However, its added role in the recruitment and activation of leukocytes may exacerbate synovial joint inflammation thereby fueling joint disease.

Though these findings are limited by lack of verification in a subsequent set of SF samples, the identification of IA-specific candidates using a label-free, MS-based approach has shown biological relevance and prospective utility for clinical applications. Future follow-up studies will address verification and validation efforts of selected protein and peptide candidates in a new set of SF and serum samples, respectively. We do acknowledge the limitation of sex discrepancy amongst the IA SF samples in our study which may have influenced the proteins and peptides identified. However, to compensate for this discrepancy between each subtype of IA, our control group consisted of an equal number of male and female SF samples. Moreover, we tested the influence of both sex and age on our data using a linear model and found there to be no effect by either predictor.

A technical limitation of this study includes the lack of fractionation of digested proteins and peptides which likely contributed to the low fold change ratios of our candidate biomarkers. Pre-fractionation methods are important for reducing the complexity of biological fluids and tissues. The proteomic profile of IA SF is markedly shifted compared to healthy SF with a greater concentration of pro-inflammatory cytokines, immunoglobulins, matrix-degrading enzymes and acute-phase markers. The dynamic range between proteins in diseased SF can vary by a factor of 10^10^ [10] and the likelihood, therefore, of masking potentially clinically-relevant proteins within the low-abundance proteome increases and may be exacerbated by analysis of unfractionated biological samples. However, improving accessibility to low-concentration proteins comes at the cost of longer analysis times and lower reproducibility [52]. Moreover, fractionation technologies have previously failed to significantly extend the sampling of the proteome relative to the unfractionated proteome [53].

## Conclusions

Chronic inflammation in IA is orchestrated by a complex network of signaling pathways which are expected to be represented in the protein and peptide expression patterns of SF. Therefore, proteomic and peptidomic analysis of SF can reflect the molecular underpinnings of IA and enhance our understanding of principal drivers at the apex of this disease. Overall, through the application of high-throughput, label-free MS, this discovery-phase study has generated a comprehensive proteomic dataset representative of IA SF and its specific subtypes. We discovered 5 protein candidates and 10 peptide candidates upregulated in IA SF, of which 3 proteins have yet to be described in IA. Moreover, subtype-specific analyses identified 4 RA-specific protein candidates, 2 PsA-specific protein candidates and 5 PsA-specific peptide candidates. Several of these candidates have been associated with inflammatory pathways at the genetic level but have not been investigated at the protein level and therefore, require functional experimentation to elucidate their role in the pathogenesis of IA. The data presented herein underscores the potential for proteins and peptides to elucidate mechanistic pathways related to the onset of arthritic disease in addition to their capacity to serve as informative clinical biomarkers.

## Additional file

**Additional file 1.** **Table S1:** Complete protein group report for proteomics. **Table S2:** Complete peptide report for proteomics. **Table S3:** Complete spectra search output for peptidomics. **Table S4:** Complete list of significantly dysregulated human proteins identified in inflammatory arthritis synovial fluid relative to control synovial fluid. **Table S5:** Complete list of significantly dysregulated human proteins identified in rheumatoid synovial fluid relative to psoriatic arthritis synovial fluid. **Table S6:** Functional pathways and regulatory networks associated with significantly dysregulated proteins in IA SF. **Table S7:** Complete list of significantly dysregulated human peptides identified in inflammatory arthritis synovial fluid relative to control synovial fluid. **Table S8:** Complete list of significantly dysregulated human peptides identified in rheumatoid synovial fluid relative to psoriatic arthritis synovial fluid. **Table S9:** Complete list of all predicted antimicrobial peptides in inflammatory arthritis synovial fluid

## Data Availability

The mass spectrometry proteomics and peptidomics datasets supporting the conclusions of this article are available in the PRIDE Archive via the PRIDE partner repository with the data set identifier PXD011872; http://www.ebi.ac.uk/pride/archive/ (username: reviewer92309@ebi.ac.uk and password: 3hXihB2 s).

## Abbreviations

IA: inflammatory arthritis
RA: rheumatoid arthritis
PsA: psoriatic arthritis
MHC: major histocompatibility complex
MS: mass spectrometry
SF: synovial fluid
AMP: antimicrobial peptide
ACR: American College of Rheumatology
CASPAR: classification criteria for psoriatic arthritis
ABC: ammonium bicarbonate
DTT: dithiothreitol
IAM: iodoacetamide
FA: formic acid
LC–MS/MS: liquid chromatography-tandem mass spectrometry
DMSO: dimethyl sulfoxide
ACN: acetonitrile
TFA: trifluoroacetic acid
LFQ: label-free quantification
IPA: ingenuity pathway analysis
DAVID: database for annotation, visualization and integrated discovery
KEGG: Kyoto Encyclopedia of Genes and Genomes
CAMP_R3_: collection of anti-microbial peptides
SVM: support vector machine
FGA: fibrinogen alpha chain
CPB2: carboxypeptidase B2
FGB: fibrinogen beta chain
F2: prothrombin
TLR: toll-like receptor
TNF-α: tumor necrosis factor alpha
IL: interleukin
CD5L: CD5 molecule-like
MMP: matrix metalloproteinase
S100: S100 calcium-binding protein
DEFA3: defensin alpha 3
FTO: alpha-ketoglutarate-dependent dioxygenase
FAM21C: family with sequence similarity 21 member C
TBX3: T-box transcription factor
GWAS: genome-wide association study
SNP: single nucleotide polymorphism
p21: cyclin-dependent kinase inhibitor p21^WAF1^
OA: osteoarthritis
FCGR3A: immunoglobulin gamma Fc region receptor III-A
POSTN: periostin
PGK1: phosphoglycerate kinase 1
COL1A1: collagen type I alpha 1
CCSER2: coiled-coil serine rich protein 2
FpA: fibrinopeptide A
ICAM-1: intercellular adhesion molecule 1
